# Scaffolds and Stem Cells Show Promise for TMJ Regeneration: A Systematic Review

**DOI:** 10.3390/bioengineering13020169

**Published:** 2026-01-29

**Authors:** Miljana Nedeljkovic, Gvozden Rosic, Dragica Selakovic, Jovana Milanovic, Aleksandra Arnaut, Milica Vasiljevic, Nemanja Jovicic, Lidija Veljkovic, Pavle Milanovic, Momir Stevanovic

**Affiliations:** 1 Department of Dentistry, Faculty of Medical Sciences, University of Kragujevac, 34000 Kragujevac, Serbia; miljananedeljkovic999@gmail.com (M.N.); jovannakg94@gmail.com (J.M.); milicavaska13@gmail.com (M.V.); veljkoviclidija008@gmail.com (L.V.); pavle11@yahoo.com (P.M.); momirstevanovic7@gmail.com (M.S.); 2 Department of Physiology, Faculty of Medical Sciences, University of Kragujevac, 34000 Kragujevac, Serbia; grosic@fmn.kg.ac.rs (G.R.); dragica984@gmail.com (D.S.); 3 Department of Histology and Embryology, Faculty of Medical Sciences, University of Kragujevac, 34000 Kragujevac, Serbia; nemanjajovicic.kg@gmail.com

**Keywords:** temporomandibular joint, temporomandibular joint disorders, temporomandibular joint disk, mandibular condyle, cartilage, tissue engineering, cartilage engineering, systematic review, stem cell, scaffolds

## Abstract

Temporomandibular joint (TMJ) disorders represent chronic degenerative musculoskeletal conditions with a high prevalence in the general population and limited regenerative treatment options. Owing to the insufficient efficacy of current conservative and surgical therapies, there is a growing clinical need for biologically based regenerative approaches. Tissue engineering (TE), particularly scaffold-based strategies, has emerged as a promising avenue for TMJ regeneration. This systematic review analyzed preclinical in vivo studies investigating scaffold-based interventions for TMJ disc and osteochondral repair. A structured literature search of PubMed and Scopus databases identified 39 eligible studies. Extracted data included scaffold composition, use of cellular and bioactive components, animal models, and reported histological, radiological, and functional outcomes. Natural scaffolds, such as decellularized extracellular matrix and collagen-based hydrogels, demonstrated favorable biocompatibility and support for fibrocartilaginous regeneration, whereas synthetic materials including polycaprolactone, poly (lactic-co-glycolic acid), and polyvinyl alcohol provided superior mechanical stability and structural tunability. Cells were used in 17/39 studies (43%); quantitative improvements were variably reported across these studies. Bioactive molecule delivery, including transforming growth factor-β, histatin-1, and platelet-rich plasma, further enhanced tissue regeneration, while emerging drug- and gene-delivery approaches showed potential for modulating local inflammation. Despite encouraging results, the reviewed studies exhibited substantial heterogeneity in experimental design, outcome measures, and animal models, limiting direct comparison and translational interpretation. Scaffold-based approaches show preclinical promise but heterogeneity in design and incomplete quantitative reporting limit definitive conclusions. Future research should emphasize standardized methodologies, long-term functional evaluation, and the use of clinically relevant large-animal models to facilitate translation toward clinical application. However, functional and biomechanical outcomes were inconsistently reported and rarely standardized, preventing robust conclusions regarding the relationship between structural regeneration and restoration of TMJ function.

## 1. Introduction

**Figure 1:**
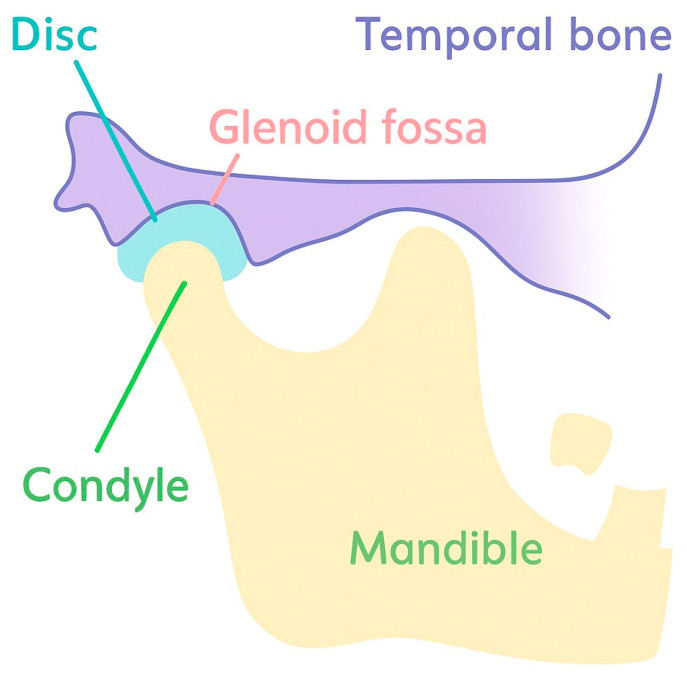
A schematic diagram of TMJ anatomy.

The temporomandibular joint (TMJ) is a synovial joint that connects the mandibular condyle with the temporal bone at the glenoid fossa, with a biconcave fibrocartilaginous articular disc interposed between them (Figure 1) [1,2,3]. This disc–condyle–fossa complex enables smooth mandibular movements under high and heterogeneous mechanical loads, while the compact anatomy and limited intrinsic healing capacity make TMJ tissues particularly susceptible to degeneration [1,2,3].

Temporomandibular disorders (TMDs) represent a group of chronic degenerative musculoskeletal conditions affecting the TMJ and associated musculature and are highly prevalent in the general population [4,5]. Their etiology is multifactorial and includes trauma, anatomical variations, and muscle hyperactivity [6,7]. Clinically, TMDs commonly manifest as joint clicking, restricted mandibular motion, myofascial pain, and headaches, while more severe symptoms such as trismus and pain during mouth opening and chewing have been linked to degenerative and osteoarthritic changes in the mandibular condyle, particularly following disc perforation [8,9]. Together, these features contribute to a substantial reduction in quality of life due to chronic pain, functional limitations, and impaired mastication [8,9].

Current treatment strategies initially rely on conservative approaches, including occlusal splints, non-steroidal anti-inflammatory drugs, lifestyle modification, exercises, and diet restrictions [8,9]. Although these modalities may relieve symptoms and slow progression in selected patients, a considerable proportion of cases ultimately require surgical intervention. Surgical options include discectomy, placement of autologous or alloplastic grafts, and total joint replacement [10,11,12,13]. However, discectomy may shift the joint toward a more degenerative state, and interposition grafting introduces additional surgery with donor-site morbidity and potential limitation of jaw motion due to scar formation; autogenous grafts have also been associated with graft resorption, functional limitations, and poor long-term outcomes [10,11,12,13]. In the absence of reliable regenerative solutions, many end-stage patients undergo total joint arthroplasty, which adds further clinical and socioeconomic burden [10,11,12,13].

Accordingly, there is a clear need for regenerative therapeutic approaches that can bridge the gap between minimally invasive treatment and terminal surgical procedures. Tissue engineering (TE) has emerged as a promising strategy for restoring TMJ integrity by combining biomaterial scaffolds with biological cues to support cell adhesion, proliferation, and extracellular matrix formation [14,15,16,17]. Nevertheless, the TMJ remains one of the most challenging anatomical targets for regeneration due to its constrained surgical accessibility, limited vascularization, and demanding mechanical environment, which together impose strict requirements on scaffold biocompatibility, degradation, and mechanical performance [14,15,16,17].

Recent preclinical work has reported regeneration of fibrocartilaginous tissues, including the TMJ disc, using scaffold-based approaches with both natural and synthetic biomaterials [18,19,20]. However, the complex structure and function of the TMJ disc and osteochondral unit require scaffolds with carefully balanced biomechanical and biological properties to withstand mandibular movements and masticatory forces, while maintaining an environment conducive to stable matrix deposition and tissue integration [8,14,15,16,17]. In addition, scaffold platforms are increasingly designed as tunable systems, allowing adjustment of biochemical and mechanical characteristics and serving as delivery vehicles for cells and growth factors [21]. Despite this rapid progress, there is still no consensus regarding which scaffold type or cell-loading strategy yields the most consistent regenerative outcomes across preclinical TMJ models [14,15,16,17].

Therefore, the aim of the present systematic review was to critically synthesize preclinical in vivo evidence on scaffold-based TE strategies for TMJ disc and osteochondral regeneration. By analyzing scaffold types, cellular and bioactive components, animal models, and reported outcomes, this review seeks to identify reproducible patterns, clarify current limitations, and inform future translational research in TMJ regeneration.

## 2. Materials and Methods

### 2.1. Study Design and Registration

This systematic review was conducted in accordance with the Preferred Reporting Items for Systematic Reviews and Meta-Analyses (PRISMA) guidelines. This systematic review was registered on the Open Science Framework (OSF) (DOI: 10.17605/OSF.IO/H3W7K). The review protocol was defined as a priori to ensure methodological transparency and reproducibility.

### 2.2. Search Strategy

A comprehensive literature search was performed in April 2025 using PubMed, Web of Science, and Scopus databases. Studies published between 1988 and April 2025 were considered eligible. The search strategy combined Medical Subject Headings (MeSH) terms and free-text keywords related to temporomandibular joint regeneration and tissue engineering, including: “temporomandibular joint” OR “TMJ” AND “tissue engineering” OR “scaffold” OR “regeneration” AND “animal model” OR “preclinical” OR “in vivo”.

The search was limited to studies published in English. All retrieved records were imported into EndNote software (version 20.5, Clarivate Analytics), where duplicate entries were identified and removed prior to screening.

### 2.3. Eligibility Criteria

Eligibility criteria were defined before study selection and are summarized in Table 1.

### 2.4. Study Selection and Data Extraction

Two authors independently screened titles and abstracts of all identified records. Full texts of potentially eligible studies were subsequently assessed for inclusion. Any disagreements were resolved through discussion until consensus was reached.

For each included study, the following data were extracted: authorship and publication year; animal species and strain; number of animals; defect type and location; scaffold material and composition; use of cells and/or bioactive molecules; follow-up duration; evaluation methods (e.g., histology, imaging, biomechanical testing); and reported outcomes. Full study-level data extraction (raw numeric values, mean ± SD where available, and timepoints) is provided as Supplementary Table S3.

### 2.5. Risk of Bias and Methodological Quality Assessment

The risk of bias and methodological quality of the included studies were independently assessed by the authors using a modified version of the SYRCLE (Systematic Review Centre for Laboratory Animal Experimentation) Risk of Bias tool, which is specifically designed for preclinical in vivo studies. The assessment included sequence generation, allocation concealment, baseline comparability, random housing, blinding of investigators and outcome assessors, completeness of outcome data, and selective outcome reporting.

Any discrepancies in assessment were resolved through group discussion. For each domain, studies were classified as having low, unclear, or high risk of bias, and the distribution of these judgments across all 39 studies was summarized using descriptive statistics and visualized as a stacked bar chart.

### 2.6. Data Synthesis and Quantitative Heterogeneity Assessment

Data were extracted in full (means, standard deviations, group sizes, and timepoints) when reported; the complete extraction is provided in Supplementary Table S1 (Excel/CSV). For outcomes reported by ≥3 studies with comparable continuous metrics we planned exploratory random-effects analyses and calculation of between-study heterogeneity (I^2^). Where continuous data or consistent metrics were unavailable, we performed quantitative summaries of data availability (number and proportion of studies reporting mean ± SD for each outcome) and descriptive subgroup counts by target tissue, animal species, defect type, scaffold class, cell/GF use and follow-up category. Pre-specified subgroup analyses/meta-regressions included animal species (rodent/rabbit/large), defect model (perforation/partial discectomy/total discectomy/osteochondral/condylectomy), scaffold class (natural/synthetic/hybrid) and follow-up (≤4 wks/4–12 wks/>12 wks). If fewer than three comparable continuous effect estimates were available for any subgroup, pooling was not performed, and the rationale is reported. The results were structured according to scaffold type, use of cellular and bioactive components, and targeted TMJ tissue (articular disc or osteochondral region).

We searched PubMed, Web of Science, and Scopus in April 2025. Articles published between 1988 and April 2025 were included in the search. The search strategy included a combination of MeSH terms and free-text keywords related to temporomandibular joint (TMJ), tissue engineering, scaffolds, growth factors, and animal models. The detailed search syntax is provided below: (“temporomandibular joint” OR “TMJ”) AND (“tissue engineering” OR “scaffolds” OR “regeneration”) AND (“animal model” OR “preclinical” OR “in vivo”).

Searches were limited to studies published in English. All identified citations were imported into EndNote software (version 20.5, Clarivate Analytics) for reference management and removal of duplicates. If fewer than three comparable continuous effect estimates were available for any subgroup, pooling was not performed, and the rationale is reported.

## 3. Results

### 3.1. Study Selection

**Figure 2:**
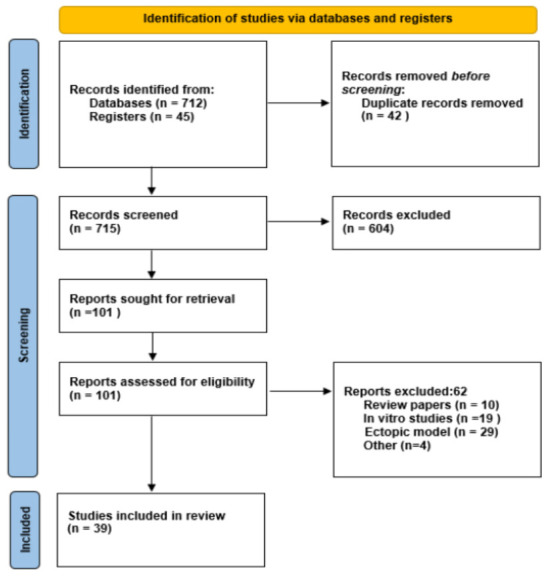
PRISMA flow diagram of study selection process.

A total of 757 records were identified, and 39 studies met all inclusion criteria and were included in the systematic review. The study selection process is illustrated in the PRISMA flow diagram (Figure 2) [22,23,24,25,26,27,28,29,30,31,32,33,34,35,36,37,38,39,40,41,42,43,44,45,46,47,48,49,50,51,52,53,54,55,56,57,58,59,60,61].

### 3.2. Characteristics of Included Studies

The 39 included studies were all preclinical in vivo trials evaluating scaffold-based tissue engineering strategies for the TMJ regeneration. Most studies used rabbits (19/39), with follow-up time from 1 week to 12 months. Other animal models included goats (6/39), rodents (6/39), dogs (3/39), sheep (3/39) and mini-pigs (1/39). Observation times varied between species: rats (2–12 weeks), rabbits (1 week to 12 months), goats (12–24 weeks) and sheep (4 months). Data availability: 22/39 studies (56%) provided at least one extractable quantitative outcome (mean ± SD or clear numeric timecourse); 17/39 (44%) provided only qualitative or semi-quantitative data (Supplementary Table S3).” “Cells were used in 17/39 studies (43%); growth factors or other bioactives were used in 14/39 studies (36%).

An overview of study characteristics is presented in Table 2.

### 3.3. Study Objectives

Of the included studies 16 evaluated TMJ disc regeneration or replacement,21 studies focused on osteochondral regeneration in the condylar region, and 2 studies investigated drug delivery systems for TMJ regeneration.

In osteochondral models, regeneration was assessed either after surgically created defects in the mandibular condyle or following condylectomy (Table 2).

### 3.4. Evaluation Methods

The diagnostic modalities used for characterizing the new bone formation included micro-CT examination, histology, immunohistochemistry, real-time PCR, fluorescence microscopy, biomechanical testing, and scanning electron microscopy.

### 3.5. Scaffold Types

All studies utilized scaffolds as the central experimental component or as delivery platforms for cells and/or bioactive substances. Most studies used synthetic polymer in the form of hydrogels (*n* = 17), in comparison to natural hydrogel scaffolds (*n* = 13) (Table 2).

### 3.6. Bioactive Molecules and Cell Therapies

Bioactive molecules were used in 14 studies (36%), either alone or in combination with a scaffold or cell therapy (Table 2). The most common growth factor was TGF-β and BMP-2. Several studies reported using various concentrations of growth factors for bone regeneration.

Cell therapies were used for TMJ regeneration in 17 studies (43%). Bone marrow stem cells (BMSCs) were mostly used to promote osteochondral regeneration. Dental-pulp-derived stem cells (DPSCs) were used in 2 studies.

A detailed overview is provided in Table 2.

Overall, the included studies demonstrate promising preclinical outcomes, but substantial methodological heterogeneity limits the ability to perform quantitative synthesis.

### 3.7. Quality Assessment of Included Studies

The methodological quality of the included animal studies was evaluated using a modified version of the Systematic Review Centre for Laboratory Animal Experimentation (SYRCLE) Risk of Bias tool, which is specifically designed to assess bias in preclinical in vivo experiments. The following domains were considered: sequence generation, allocation concealment, baseline characteristics, blinding of investigators and outcome assessors, random housing, incomplete outcome data, and selective outcome reporting.

**Figure 3:**
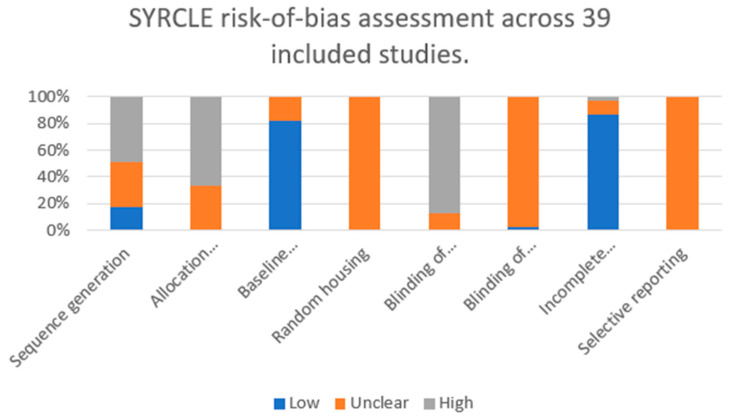
Domain-level risk-of-bias assessment of the 39 included preclinical in vivo studies according to the SYRCLE tool. Bars represent the proportion of studies rated as low (blue), unclear (orange), or high (grey) risk of bias for each domain (sequence generation, allocation concealment, baseline comparability, random housing, blinding of investigators, blinding of outcome assessors, incomplete outcome data, and selective reporting).

For each of the eight SYRCLE domains, we quantified the number and proportion of studies judged as low, unclear, or high risk of bias (Figure 3). Across the 39 included studies, sequence generation (randomization) was most frequently rated as high risk (19/39, 48.7%), with only 7 studies (17.9%) judged as low risk and 13 (33.3%) as unclear. Allocation concealment showed an even less favorable profile, with no study meeting low-risk criteria (0/39, 0%), while 13 (33.3%) were classified as unclear and 26 (66.7%) as high risk.

In contrast, baseline comparability between experimental groups was predominantly adequate, with 32/39 studies (82.1%) rated as low risk and 7/39 (17.9%) as unclear, and none classified as high risk. Random housing was not explicitly reported in any of the studies and was therefore uniformly judged as unclear risk (39/39, 100%).

Blinding of investigators was frequently rated as high risk (34/39, 87.2%). Blinding of outcome assessors was almost never clearly reported: 1/39 (2.6%) low risk, 38/39 (97.4%) unclear.

Conversely, incomplete outcome data represented a methodological strength of the included literature, with 34/39 studies (87.2%) judged as low risk, 4/39 (10.3%) as unclear, and only 1/39 (2.6%) as high risk. Selective outcome reporting could not be reliably assessed due to the lack of preregistered protocols; all 39 studies (100%) were therefore rated as unclear risk in this domain.

Taken together, these findings indicate that the most frequently violated or insufficiently reported domains were allocation concealment and blinding of investigators, whereas baseline comparability and completeness of outcome data were generally well addressed. Detailed descriptions of study designs, animal models, scaffold types, and interventions for each of the 39 included studies are shown in Table 2, whereas the aggregated domain-level risk-of-bias judgments are summarized in Table 3 and Figure 3.

Study-level risk-of-bias judgments for each domain are provided in Supplementary Table S2.

### 3.8. Heterogeneity Assessment and Data Availability

The full extracted dataset is provided as Supplementary Table S3. Overall, 22/39 studies (56%) reported at least one extractable quantitative outcome (mean ± SD or clear numeric time-course); 17/39 (44%) provided only qualitative or semi-quantitative data. Summary counts (see Supplementary Table S3):

Target tissue: Disc 16, Osteochondral/condyle 21, Drug delivery 2.

Animal groups: Small rodents 6, Rabbits 19, Large animals (goat/sheep/pig/dog) 14.

Scaffold class: Synthetic 17, Natural 13, Hybrid 9.

Studies reporting cells: 17/39 (43%); reporting growth factors/bioactives: 14/39 (36%). When we inspected specific outcomes (histological scores, μCT bone metrics, biomechanical tests), fewer than three studies reported the same continuous outcome metric within the same tissue/animal/defect subgroup for most comparisons. For example, only two rabbit osteochondral GelMA studies reported comparable μCT BV/TV metrics. Biomechanical outcomes with mean ± SD were present in only five studies across heterogeneous species and tests. We therefore constructed a data-availability matrix (Supplementary Table S3) listing the number of studies with extractable mean ± SD for each outcome by subgroup; this matrix documents the precise gaps that precluded reliable pooled analyses. Exploratory meta-analysis was planned a priori but no prespecified subgroup met the *n* ≥ 3 and metric-consistency criteria for a primary pooled analysis; where *n* ≥ 3 homogeneous estimates are identified later, these data are provided to enable future pooling.

### 3.9. Functional and Biomechanical Outcomes

Across the 39 included studies, functional and biomechanical assessment of regenerated TMJ tissues was inconsistently reported. Eighteen of 39 studies (≈46%) described at least one functional or biomechanical outcome (e.g., joint kinematics, mastication-related behavior, or ex vivo mechanical testing of regenerated tissues).

Where reported, functional outcomes included joint kinematics (e.g., range and symmetry of mandibular motion) in large-animal disc replacement models, qualitative or semi-quantitative assessments of chewing behavior, and, more rarely, measurements related to jaw opening or mastication patterns in rodent models of TMJ inflammation. Biomechanical testing most commonly involved ex vivo measurement of compressive or tensile properties of native or regenerated discs and osteochondral constructs (e.g., elastic modulus, stiffness, failure load), comparison of the mechanical behavior of decellularized or synthetic scaffolds to native tissues, or time-dependent changes in scaffold/tissue mechanical performance.

However, the specific parameters, testing protocols, and reporting formats varied substantially between studies. For most combinations of tissue type, animal model, and defect configuration, fewer than three studies reported the same biomechanical metric with comparable methodology, and standardized functional endpoints such as maximal interincisal opening, lateral excursions, bite force, or quantitative mastication efficiency were rarely measured. As a result, we were unable to perform pooled analyses of functional restoration or to formally test correlations between functional and structural (histological or radiological) regeneration.

## 4. Discussion

This systematic review synthesizes preclinical evidence on scaffold-based tissue engineering (TE) strategies for regeneration of temporomandibular joint (TMJ) structures [16,61,62,63,64]. Overall, the findings indicate that scaffold-based approaches can support disc and osteochondral regeneration under experimental conditions; however, regenerative success is highly dependent on scaffold design, biological augmentation, and model-specific factors. Importantly, while numerous studies report favorable histological outcomes, robust functional restoration and long-term durability remain insufficiently demonstrated, underscoring the persistent translational gap between experimental feasibility and clinically reliable TMJ regeneration [16,26]. In our dataset, functional and biomechanical outcomes were reported in less than half of the included studies, and even when present, they were rarely standardized or quantitatively comparable. Only a small subset of studies provided mean ± SD values for biomechanical properties (e.g., tensile or compressive modulus, stiffness, failure load) or for clinically relevant functional surrogates such as jaw kinematics or mastication-related behavior. Moreover, no study systematically combined quantitative functional measurements (e.g., maximal interincisal opening, lateral excursions, bite force, or mastication efficiency) with detailed structural assessment in a way that would allow robust evaluation of whether functional restoration tracks with histological or radiological regeneration. This fragmented and heterogeneous reporting represents a critical analytical gap and likely explains why encouraging structural findings have not yet translated into predictable clinical functional benefits.

### 4.1. TMJ Articular Disc Regeneration

Among TMJ components, the articular disc has received particular attention due to its essential biomechanical role and limited intrinsic healing capacity [1,2,3,4,5]. Across the included studies, both natural and synthetic scaffolds demonstrated the ability to support fibrocartilaginous regeneration, although outcomes were variable and strongly influenced by scaffold composition and experimental models.

Natural scaffolds, particularly decellularized extracellular matrix (ECM) and collagen-based hydrogels, consistently exhibited favorable biocompatibility and integration with surrounding tissues. Several studies reported formation of fibrocartilage-like tissue with improved cellular organization and extracellular matrix deposition following implantation of ECM-derived or collagen-based constructs, especially in rabbit, canine, and goat models [22,24,27,31,32,33,34,35,36]. Nevertheless, the limited mechanical strength of natural scaffolds, together with variable degradation rates and potential immunogenicity, remains a critical limitation in the load-bearing TMJ environment [14,16,17].

Synthetic scaffolds offered improved reproducibility and tunable mechanical properties. Materials such as polyvinyl alcohol (PVA), polycaprolactone (PCL), and poly(lactic-co-glycolic acid) (PLGA) demonstrated enhanced resistance to joint loading and more predictable degradation behavior [23,25,26]. Importantly, composite scaffolds incorporating controlled release of growth factors, such as connective tissue growth factor and transforming growth factor-β, resulted in superior regenerative outcomes compared with scaffold-only approaches [29,37]. These findings emphasize the importance of biomimetic scaffold design and spatiotemporal bioactive signaling. In contrast, purely synthetic constructs lacking biological augmentation frequently failed to achieve complete functional restoration and, in some cases, induced degenerative changes [25].

Disc regeneration outcomes were also strongly influenced by the defect model. Studies employing disc perforation models differed substantially from those using partial or total discectomy, while spontaneous healing varied across animal species [29,34]. The absence of clearly defined critical-size defect models for TMJ disc injury represents a major limitation in the current literature and hinders direct comparison between studies [29,34].

Taken together, available preclinical data suggest that neither natural nor synthetic scaffolds alone consistently ensure predictable TMJ disc regeneration; this interpretation is limited by heterogeneous and largely non-comparable outcome reporting (see Supplementary Table S3). Hybrid constructs that combine mechanical stability with controlled biological signaling appear most promising; however, the lack of standardized defect models and functional outcome measures precludes definitive conclusions regarding optimal scaffold design.

### 4.2. Osteochondral Regeneration of the Mandibular Condyle

Osteochondral regeneration of the mandibular condyle represents an equally complex challenge in TMJ tissue engineering [2,3,4,5,6]. Injectable hydrogels have emerged as particularly attractive platforms due to their minimally invasive application, adaptability to irregular articular surfaces, and capacity for local delivery of cells and bioactive agents [65,66,67,68,69,70,71,72,73,74,75].

Multiple studies demonstrated that scaffolds seeded with mesenchymal stem cells (MSCs), particularly bone marrow-derived MSCs, significantly enhanced cartilage and subchondral bone regeneration [43,44,46,47,50,76,77,78]. Improved outcomes were generally attributed to enhanced chondrogenic differentiation, modulation of the inflammatory microenvironment, and improved integration with host tissue. However, regenerative efficacy varied across studies, and inconsistent results—particularly those involving alternative stem cell sources such as dental pulp stem cells [79]—suggest that successful regeneration depends on complex interactions between cell type, scaffold properties, and local mechanical conditions [38,42].

Biofunctionalization of scaffolds with growth factors or signaling peptides further improved osteochondral repair. Histatin-1-functionalized scaffolds and platelet-rich plasma–enriched hydrogels promoted angiogenesis, cell recruitment, and matrix remodeling in several models [39,40,50]. In contrast, the role of potent osteogenic factors such as bone morphogenetic protein-2 remains controversial, as inconsistent cartilage outcomes and the risk of ectopic ossification highlight the need for precise control of dosing, localization, and release kinetics [53,55,57].

Collectively, these findings indicate that osteochondral regeneration of the mandibular condyle is feasible in preclinical models, particularly when cell-based strategies are employed. Nevertheless, inconsistent outcomes across studies demonstrate that biological augmentation cannot compensate for suboptimal scaffold mechanics or inadequate experimental design.

### 4.3. Comparative Performance of Cell-Based and Acellular Approaches

Cells were used in 17/39 studies (43%), most frequently bone marrow–derived mesenchymal stem cells (BMSCs), followed by adipose-derived stem cells, dental pulp stem cells (DPSCs), and chondrocytes. Across osteochondral and cartilage models, BMSC-seeded scaffolds generally showed improved histological scores, more mature cartilage and subchondral bone, and in some cases better μCT parameters compared with acellular scaffolds. However, reporting of continuous outcomes (mean ± SD) was inconsistent, and few studies used identical scoring systems, which precluded quantitative pooling of effect sizes. DPSCs were used in only two studies, both suggesting early benefits on cellularity and matrix deposition but without sufficient, standardized outcome data to compare their efficacy directly with BMSCs. Overall, the current evidence supports the use of MSC-based strategies over acellular scaffolds in terms of qualitative regeneration, but the small number of homogeneous datasets and heterogeneous outcome measures prevents robust ranking of specific cell sources.

### 4.4. Scaffold-Based Drug Delivery Strategies

Scaffold-based drug delivery systems have emerged as an innovative approach for addressing the inflammatory component of temporomandibular disorders [59,60]. Compared with conventional intra-articular injections, hydrogel-based delivery platforms enable sustained local release while potentially reducing the need for repeated interventions. Preclinical studies utilizing siRNA-loaded PLGA microparticles or naproxen-loaded lipid carriers demonstrated effective suppression of inflammatory cytokines and reduced leukocyte infiltration in experimental TMJ models [59,60]. Although these strategies primarily target inflammation rather than structural regeneration, they may serve as valuable adjuncts within broader regenerative treatment paradigms.

### 4.5. Growth Factors, Bioactive Molecules, and Delivery Strategies

Growth factors and other bioactive molecules were incorporated in 14/39 studies (36%), most commonly transforming growth factor-β (TGF-β), bone morphogenetic protein-2 (BMP-2), histatin-1, NELL-1, fibroblast growth factor-2 (FGF-2), and platelet-rich plasma (PRP). In general, biofunctionalization of scaffolds with these agents improved histological appearance of cartilage and subchondral bone, as well as μCT and molecular readouts, when compared with scaffold-only controls. Histatin-1–functionalized GelMA scaffolds consistently enhanced osteochondral repair quality relative to nonfunctionalized GelMA, and PRP-enriched hydrogels promoted more complete osteochondral fill and favorable macrophage polarization in a rabbit model.

In contrast, studies using BMP-2 reported robust osteogenesis but variable effects on articular cartilage, with concerns about ectopic ossification and irregular subchondral remodeling. However, the small number of BMP-2 studies and heterogeneous dosing regimens, defect models, and outcome measures did not allow us to quantify the frequency of such complications or to define an optimal dose window. Similarly, because different growth factors were almost never compared head-to-head within the same model using standardized metrics, it was not possible to establish the relative efficacy of TGF-β, BMP-2, NELL-1, PRP, or histatin-1 using pooled effect estimates.

Delivery strategies for bioactive molecules varied substantially and included simple adsorption or mixing with hydrogels (bolus release), encapsulation in microparticles or injectable hydrogels for sustained release, and gradient delivery within multilayered constructs. Although studies employing controlled or sustained-release systems often reported more stable cartilage and bone regeneration than those relying on bolus delivery, the number of studies per combination of factor, carrier, and defect model was too low to formally test whether delivery mode explained outcome variability. Consequently, we provide a structured narrative synthesis instead of a comparative quantitative ranking of individual growth factors or delivery systems [61,62,63,64].

### 4.6. Animal Models and Translational Considerations

The choice of animal model substantially influenced reported outcomes across studies. Small-animal models provided important mechanistic insights but exhibited limited translational relevance due to anatomical and biomechanical differences from the human TMJ [80,81,82,83,84,85,86]. Larger animal models, including goats, sheep, dogs, and mini-pigs, more closely approximated human TMJ anatomy and loading conditions, enabling assessment of scaffold fixation, durability, and functional performance [22,25,35,36,41]. However, ethical, logistical, and economic constraints continue to limit their widespread use [86,87,88,89]. The 39 included studies used a wide spectrum of animal models (rats, rabbits, goats, sheep, dogs, and mini-pigs), which differ substantially in TMJ anatomy, biomechanical loading, and intrinsic healing capacity. These interspecies differences are crucial for interpreting regenerative outcomes and for selecting appropriate preclinical models.

Rodents and rabbits have been valuable for early mechanistic and proof-of-concept studies because of their low cost and ease of handling. However, many small laboratory animals experience relatively low TMJ loads during mastication and display joint orientations and movement patterns that differ from humans, limiting their ability to replicate the complex fibrocartilaginous loading environment of the human disc [25,85,87]. In addition, the small disc size in rodents and rabbits constrains surgical access, fixation strategies, and the use of anatomically realistic implants.

In contrast, large-animal models such as sheep, goats, mini-pigs, and dogs more closely approximate human TMJ anatomy, disc dimensions, and loading conditions [25,90,91]. Detailed morphologic, histological, and biomechanical characterization of the ovine TMJ disc has demonstrated notable similarities to the human disc, including an elliptical, biconcave fibrocartilaginous structure, a thinner central zone supported by a thicker peripheral “ring-like” region, abundant collagen and elastic fibres, and tensile and compressive moduli in the same order of magnitude as reported for human discs [25,25,85,90]. The relative position of the disc between the mandibular condyle and glenoid fossa, the separation into upper and lower joint compartments, and the relationship to adjacent structures (external acoustic meatus, foramen ovale) are also comparable [25,25,90,92]. At the same time, important differences must be acknowledged: in sheep, mediolateral mandibular movements predominate, the condylar surface is mediolaterally concave, and the temporal fossa is comparatively flat, more closely resembling the edentulous human TMJ [25]. These features highlight that no single animal model fully reproduces the human joint, and species-specific biomechanics need to be considered when extrapolating preclinical findings.

Our dataset reflects this range of models: 6 small-rodent, 19 rabbit, and 14 large-animal studies (goat, sheep, mini-pig, dog). When outcomes were examined by species, large-animal studies were more likely to report clinically relevant parameters such as implant fixation, long-term degenerative changes, and functional measures of mastication and jaw motion, whereas small-animal studies predominantly reported short- to medium-term histology and imaging. In a randomized ovine trial of three interposal disc implants, poly(ε-caprolactone) (PCL) and PCL + poly(ethylene glycol) diacrylate (PCL + PEGDA) devices led to pronounced condylar osteolysis, loss of non-hyaline cartilage, foreign-body reactions and osteoarthritic changes, whereas a poly(glycerol sebacate)/PCL (PGS + PCL) elastomeric scaffold was completely resorbed within 6 months and largely preserved articular cartilage and subchondral bone architecture [26]. These results illustrate that materials appearing promising on the basis of mechanical testing or small-animal data may fail under the higher and more complex loading conditions present in large-animal TMJs.

Taken together, the available evidence supports a tiered approach to model selection. Small rodents and rabbits are appropriate for early-stage studies aimed at screening scaffold compositions, cell and drug-delivery strategies, and basic mechanisms of TMJ regeneration. However, evaluation of fixation methods, long-term durability, scaffold degradation, subchondral remodelling and osteoarthritic sequelae should preferentially be performed in large-animal models (sheep, goats, mini-pigs) that more closely reproduce human TMJ anatomy and functional loading [25,25,26,87,90,93]. For disc replacement, medium-term rabbit studies (3–6 months) can be used to refine implant design and biological augmentation, but at least one long-term large-animal study (≥6–12 months) with quantitative imaging, histology and, where feasible, kinematic assessment of mastication and jaw opening appears necessary before considering clinical translation. For condylar osteochondral repair, similar follow-up durations in large animals are likely required to capture subchondral bone remodelling and potential implant-related degeneration.

Methodological limitations were common among the included studies and are clearly reflected in the domain-level SYRCLE assessment. Nearly half of the studies were judged to have a high risk of bias for sequence generation (19/39, 48,7%). Our analysis has shown that the lack of clearly described randomization and blinding represents one of the most significant sources of bias across the included preclinical studies, as demonstrated by the SYRCLE assessment (Figure 3, Table 3). Insufficient reporting and implementation of these methodological safeguards substantially limits internal validity and reduces the translational reliability of the reported regenerative outcomes. Therefore, future preclinical TMJ tissue engineering studies should prioritize standardized study designs with explicit reporting of randomization and blinding procedures. Inadequate reporting of randomization and blinding, lack of standardized defect models, and limited use of biomechanical testing likely contributed to variability in reported outcomes [16]. Furthermore, control strategies were heterogeneous across the included studies. As summarized in Supplementary Table S3, several studies used the contralateral TMJ of the same animal as the primary control, whereas others relied on independent control animals or defect-only/no-scaffold controls. In unilateral models, use of the contralateral joint as a control may underestimate or distort the effect of the intervention, because altered loading and compensatory mastication can induce secondary changes in the non-operated TMJ; such contralateral degeneration has been documented in experimental models of unilateral TMJ injury [94]. These observations indicate that contralateral TMJs do not necessarily represent “healthy” or unbiased controls over time, and future preclinical TMJ regeneration studies should therefore clearly report the type of control used and, where feasible, favor bilateral intervention models or separate control cohorts [95,96] (see Supplementary Table S3).

We quantified data availability prior to any pooling attempt. Heterogeneity derived from: (i) divergent animal species and scale (rodent vs. rabbit vs. large animals), (ii) multiple defect models (perforation/partial discectomy/total discectomy/osteochondral/condylectomy), (iii) wide follow up range (1 week–12 months), and (iv) non standardized outcome metrics (multiple histological scoring systems, different μCT measures, and disparate biomechanical tests). Among these sources of heterogeneity, interspecies differences in TMJ anatomy, loading patterns, and healing capacity between small and large animals are likely to be particularly important for explaining variability in regenerative outcomes. Key drivers of heterogeneity included animal species, defect model, scaffold class, follow-up duration, and non-standardized outcome metrics; details on growth factor concentrations, dosing schedules, and per-group sample sizes were frequently missing, preventing standardized comparative analyses. Despite encouraging histological and radiological findings reported across multiple studies, substantial heterogeneity prevented formal pooling of effect sizes. Our Supplementary Table S3 (data-availability matrix) documents that for most clinically relevant outcomes there were <3 studies reporting the same continuous metric within a homogeneous subgroup. This lack of comparable effect estimates prevented robust pooled effect calculation and reliable heterogeneity statistics (I^2^). Therefore, we report a transparent narrative synthesis supported by the Supplementary Table S3 to enable reproducibility and facilitate future meta-analysis when standardized data become available.

### 4.7. Translational Perspective and Clinical Relevance

Despite encouraging preclinical outcomes, translation of scaffold-based tissue engineering strategies for temporomandibular joint regeneration into clinical trials remains limited. Key challenges include ensuring material safety, sterilizability, and reproducibility, as well as compliance with regulatory requirements and scalable manufacturing processes [94,97,98,99]. In practical terms, acellular TMJ scaffolds intended as implants are likely to follow device pathways (e.g., 510(k) or Investigational Device Exemption in the United States, or CE marking under the European Medical Device Regulation), whereas cell-loaded or gene-modified constructs would typically fall under advanced therapy medicinal product (ATMP) frameworks. These categories imply different expectations regarding preclinical evidence, manufacturing under good manufacturing practice (GMP) conditions, and the design of early-phase clinical trials. In addition, most preclinical studies lack long-term functional assessment and biomechanical validation under physiologically relevant loading conditions, which are essential prerequisites for clinical application [100].

Cell-based approaches, particularly those involving mesenchymal stem cells, have demonstrated short-term safety in intra-articular applications across various musculoskeletal indications, including osteoarthritis [101,102,103]. However, evidence for durable structural regeneration remains inconclusive. In the context of temporomandibular joint disorders, clinical data is scarce and largely limited to exploratory or small-scale studies. Notably, intra-articular injection of bone marrow-derived nucleated cells has been reported to improve clinical symptoms in patients with temporomandibular disorders, although radiological evidence of cartilage regeneration was not observed during follow-up [104]. Similar limitations have been described in later-stage clinical trials evaluating mesenchymal stem cell therapies for joint cartilage repair, where symptomatic improvement was not consistently accompanied by structural regeneration [105,106,107,108,109,110,111]. Taken together, these observations suggest that preclinical endpoints focused predominantly on histological or imaging markers of structural regeneration may not fully predict clinically meaningful improvements in pain, jaw function, or quality of life, and that functional outcome measures in animal models should be selected with these clinical endpoints in mind.

### 4.8. Implications for Future Research and Clinical Translation

In summary, scaffold-based tissue engineering strategies demonstrate clear regenerative potential for TMJ structures in preclinical settings, particularly when mechanically competent scaffolds are combined with mesenchymal stem cells and controlled bioactive signaling. However, current evidence remains insufficient to support direct clinical translation. Future progress will depend on standardized defect models, rigorous biomechanical validation, and long-term functional assessment in clinically relevant animal models. In the future perspectives, we emphasize that the evaluation of regeneration must not remain solely at the histological level. It is essential that future studies include standardized biomechanical tests that assess load-bearing capacity, functional performance, and long-term stability of the regenerated tissue. Only through a combination of histological, biomechanical, and functional outcomes can truly translational results in TMJ regeneration be achieved. Without such methodological refinement, promising experimental outcomes are unlikely to translate into predictable therapeutic solutions for patients with temporomandibular disorders. Based on the available data, we suggest that clinically oriented preclinical programs may include medium-term rabbit studies (3–6 months) and, where feasible, at least one long-term large-animal model (6–12 months) for disc replacement or condylar osteochondral repair, together with a basic set of functional outcomes (e.g., maximal interincisal opening and quantitative measures of mastication or bite force) and biomechanical testing of regenerated tissues under relevant loading. In parallel, clearer reporting of key manufacturing parameters and brief consideration of the intended regulatory route (device versus ATMP) could help better align experimental designs with subsequent clinical development. To address these gaps, we propose that future preclinical TMJ regeneration studies include a basic standardized panel of functional and biomechanical endpoints. At the joint level, this should at least comprise maximal interincisal opening and lateral/protrusive mandibular excursions (in mm), together with objective mastication-related measures such as bite force or mastication efficiency. At the tissue level, regenerated discs and osteochondral constructs should undergo ex vivo mechanical testing, including tensile and/or compressive modulus, stiffness and failure load, and, where feasible, cyclic loading under physiologically relevant conditions. These outcomes should be reported with clear test protocols and mean ± SD with group sizes, to enable both within-study interpretation and cross-study comparison. Harmonized functional and biomechanical outcome protocols would allow assessment of whether histologically promising scaffold-based strategies truly restore TMJ function under masticatory loading.

## 5. Conclusions

This systematic review provides a comprehensive synthesis of preclinical in vivo evidence on scaffold-based tissue engineering strategies for regeneration of temporomandibular joint structures. The available data demonstrates that both disc and osteochondral regeneration can be achieved under experimental conditions, particularly when mechanically competent scaffolds are combined with mesenchymal stem cells and carefully controlled bioactive signaling.

Scaffold-based approaches show preclinical promise, but translation is limited by heterogeneous study designs and incomplete quantitative reporting. Standardized reporting of sample sizes, primary outcome means ± SD, timepoints, and adverse events is required to enable robust comparative analyses. Importantly, functional restoration and long-term durability remain insufficiently addressed in most preclinical investigations, with fewer than half of the available studies reporting functional or biomechanical endpoints and very few using standardized, quantitatively comparable protocols. This lack of systematic functional evaluation constitutes a major barrier to translation, because histological and radiological improvements cannot, by themselves, guarantee clinically meaningful restoration of TMJ function.

Current evidence suggests that scaffold-based approaches alone are unlikely to ensure predictable TMJ regeneration. Instead, integrated strategies that balance mechanical stability with biological augmentation appear most promising. However, successful translation will depend on methodological refinement, including standardized defect models, rigorous biomechanical evaluation, and long-term functional assessment in clinically relevant animal models. Standardized reporting of cell type, dose, and delivery parameters, as well as growth factor identity, concentration, and release kinetics, will be essential to identify which combinations yield the most consistent and clinically relevant regenerative effects. In addition to standardized outcome reporting, model selection will be critical for successful translation. Small-animal experiments are appropriate for early proof-of-concept work, but positive findings in rodents or rabbits should be confirmed in at least one clinically relevant large-animal model that reproduces human TMJ anatomy and loading conditions before progression to human trials.

We emphasize that large animal models, such as sheep, goats, or minipigs, are essential due to their anatomical and biomechanical similarity to the human TMJ. They enable the assessment of long-term stability, fixation, and functional performance under human-like conditions, and therefore represent a crucial step toward clinical application.

In conclusion, scaffold-based tissue engineering represents a compelling regenerative concept for temporomandibular joint disorders, but its clinical implementation remains premature. Future well-designed preclinical studies are essential to establish reproducible, functionally relevant outcomes and to provide a robust foundation for eventual translation into clinical trials.

## Figures and Tables

**Table 1:** Eligibility Criteria for Study Selection - Inclusion and Exclusion Criteria.

Criteria	
Including criteria	Original in vivo studies using animal models for orthotopic regeneration of TMJ structures; Use of natural and/or synthetic scaffolds; Clear description of surgical procedures, scaffold composition, and evaluation methodology; Inclusion of at least one experimental and one control group; Defined follow-up period and histological and/or radiological outcome assessment.
Excluding criteria	Review articles, editorials, letters, and conference abstracts; In vitro studies and ectopic implantation models; Studies lacking sufficient methodological or outcome data.

**Table 2:** Overview of revised articles.

Ref.	First Author (Year)	Target Tissue	Animal Species	Defect/Model	Orthotopic/Ectopic	Scaffold Class	Cells Used	GF/Bioactive	Follow-Up	Usable Quantitative Outcomes
[22]	Socorro 2025	Disc	Goat	Total vs. partial discectomy + SIS ECM	Orthotopic	Natural ECM sheet (SIS)	No	No	≤4 weeks	No—qualitative histology only
[23]	Gan 2022	Disc	Rabbit	Disc defect/replacement	Orthotopic + ectopic	Synthetic 3D scaffold (PCL/PLA/CNT)	No (in vivo)	No	≤4 weeks	Limited—semi-quantitative histology
[24]	Liang 2020	Disc (material)	Pig	Material characterization	Ectopic/in vitro	Natural dECM hydrogel	Yes (in vitro)	No	≤1 week	Yes—DNA, GAG, COL, modulus
[25]	Angelo 2016	Disc	Sheep	Native disc characterization	N/A	None	N/A	N/A	N/A	Yes—mechanical properties
[26]	Angelo 2021	Disc replacement	Sheep	Bilateral total discectomy	Orthotopic	Synthetic/hybrid PCL	No	No	>12 weeks	Yes—CT & histology scores
[27]	Jiang 2023	Disc	Rabbit/goat	Disc resection	Orthotopic	Natural dECM ± PCL	No	No	>12 weeks	Yes—mechanical comparison
[28]	Chung 2022	Disc	Dog	Total meniscectomy	Orthotopic	Natural SIS ECM	No	No	≤4 weeks	Yes—GLP histology, MRI
[29]	Moura 2020	Disc	Sheep	Bilateral discectomy	Orthotopic	Synthetic/hybrid	No	No	>12 weeks	Yes—CT, kinematics
[30]	Tarafder 2016	Disc/meniscus	Rabbit	TMJ platform models	Orthotopic + ectopic	Synthetic PCL	Yes (MSCs)	Small molecules	≤4 weeks	Limited TMJ quantitative data
[31]	Xu 2022	Disc + TMJOA	Mini-pig/rat	Disc perforation/OA	Orthotopic	Natural DN hydrogel	No	Intrinsic	4–8 weeks	Yes—mechanics, imaging
[32]	Wang 2017	Disc	Rabbit	Partial defect	Orthotopic	Natural collagen	No	No	1–3 months	Partly—inconsistent data
[33]	Chan 2004	Disc	Rabbit	Partial defect	Orthotopic	Natural collagen	No	No	3 months	No—qualitative
[34]	Lai 2005	Disc	Rabbit	Partial discectomy	Orthotopic	Natural collagen	No	No	1–3 months	No—descriptive
[35]	Kobayashi 2015	Disc	Rabbit	Disc perforation	Orthotopic	Natural collagen	Yes (auto marrow)	No	2–8 weeks	No—qualitative
[36]	Brown 2011	Disc	Dog	Meniscectomy	Orthotopic	Natural UBM ECM	No	No	3–24 weeks	Yes—cell density, vessels
[37]	Brown 2012	Disc	canine ( female mongrel dogs, 15–20 kg)	bilateral TMJ disectomy; unilateral disc replacement	Orthotopic	ECM-based ( UBM, particulate + sheet multilaminate )	None added ( host cells infiltrate scaffold)	Endogenous bioactive cues in UBM	6 months	Yes-mechanical and biochemical
[38]	Ahtiainen 2013	Disc replacement	Rabbit	Total disc removal	Orthotopic	Synthetic PLA	Yes (ASCs)	TGF-β1	6–12 months	Limited numeric data
[39]	Monteiro 2024	Condyle cartilage	Rabbit	Osteochondral defect	Orthotopic	Synthetic GelMA	±DPSCs	No	≤4 weeks	Yes—μCT, histology
[40]	Du 2023	Condyle osteochondral	Rabbit	TMJ defect	Orthotopic	Synthetic GelMA	No	Histatin-1	1–4 weeks	Yes—quantitative
[41]	Shi 2021	Condyle osteochondral	Rabbit	TMJ defect	Orthotopic	Synthetic GelMA	No	Histatin-1	1–4 weeks	Yes—time-course
[42]	Nedrelow 2023	Condyle prosthesis	Goat	Segmental condylectomy	Orthotopic	Synthetic PCL-HAp	No	No	6 months	Yes—imaging/mechanics
[43]	Ma 2024	Condyle cartilage	Rat	Cartilage defect	Orthotopic	Synthetic ROS hydrogel	±DPSCs	ROS-responsive	≤1 week	Yes—apoptosis markers
[44]	Wang 2021	Condyle osteochondral	Rabbit	Osteochondral defect	Orthotopic	Hybrid collagen/BCP	Yes (rBMSCs)	No		Yes—μCT, histology
[45]	Yu 2021	Condyle osteochondral	Goat	Osteochondral defect	Orthotopic	Synthetic hydrogel	Chondrocytes ± BMSCs	No	4–12 weeks	Yes—histology
[46]	Guo 2022	Articular cartilage	Rat	Knee defect	Orthotopic	Synthetic hydrogel	No	No	≤4 weeks	Yes—mechanics
[47]	Yang 2022	Condyle cartilage	Rat	TMJOA	Orthotopic	GelMA microspheres	Yes (rBMSCs)	TGF-β	≤2 weeks	Yes—qPCR, μCT
[48]	Sun 2018	Condyle cartilage	Goat	Full-thickness defect	Orthotopic	Synthetic hydrogel	Chondrocytes + BMSCs	No	4–12 weeks	Semi-quantitative
[49]	Zhang 2018	Articular cartilage	Rabbit	Knee defect	Orthotopic	Synthetic thermogel	Yes (BMMSCs)	No	12 weeks	Yes—modulus, GAG
[50]	Wang 2021	TMJOA	Rabbit	Collagenase OA	Orthotopic	Synthetic injectable	No	Nell-1	6 weeks	Yes—μCT
[51]	Jiang 2021	Osteochondral	Rabbit	Femur defect	Orthotopic	Synthetic GelMA	No	PRP	6–18 weeks	Yes—ICRS, μCT
[52]	Yu 2011	Condyle cartilage	Goat	Segmental condylar osteotomy with transport disc	Orthotopic	Hydrogel ( Pluronic F -127)	Autologous chondrocytes	Endogenous (chondrocytes)	12 weeks	Yes- histology and immunohistochemistry
[53]	Zhu 2011	Condyle osteochondral	Goat	Large TMJ defect	Orthotopic	Synthetic PLGA	Yes (BMSCs)	NELL-1	6–24 weeks	Yes—histology, μCT
[54]	Dormer 2011	Condyle osteochondral	Rabbit	1 × 3 mm defect	Orthotopic	Synthetic PLGA	No	BMP-2 + TGF-β1	6 weeks	Yes—scores
[55]	Takafuji 2007	Condyle cartilage	Rabbit	Cartilage defect	Orthotopic	Natural collagen	No	FGF-2	3 weeks	Partly—categorical
[56]	Suzuki 2002	Condyle cartilage	Rabbit	Cartilage defect	Orthotopic	Natural collagen	No	BMP-2	3 weeks	Partly—qualitative
[57]	El-Bialy 2010	Full TMJ	Rabbit	Total condylectomy	Orthotopic	Natural UBM ECM	Yes (BMSCs)	LIPUS	4 weeks	Yes—qPCR, μCT
[58]	Ueki 2003 (PGS + BMP)	Condyle osteochondral	Rabbit	Condylectomy	Orthotopic	Synthetic PGS	No	rhBMP-2	2–24 weeks	Yes—trends
[59]	Ciocca 2013	Condyle osteochondral	Sheep	Mandibular condyle replacement with CAD-CAM HA scaffold	Orthotopic	Porous hydroxyapatite (HA)	MSCs (bone marrow-derived)	Platelet-rich plasma (PRP)	16 weeks	Yes- histomorphometry, bone ingrowth, porosity, statistical comparasion
[60]	Mountiziaris 2012	TMJ (inflammation)	Rat	CFA-induced TMJ inflammation model	Orthotopic	Synthetic polymeric microparticles (PLGA MPs)	No	siRNA (anti-FcγRIII)	≤9 days	Yes—quantitative meal pattern analysis, cytokine expression (IL-1β, IL-6), protein knockdown levels
[61]	Guilherme 2019.	TMJ (inflammation)	Rat	Chemically induced TMJ inflammation	Orthotopic	Natural lipid-based carrier (nanostructured lipid carrier)	No	Naproxen	Short-term (days)	Yes—quantitative histological inflammation scores and biochemical inflammatory markers

**Table 3:** SYRCLE risk-of-bias summary.

Domen	Low Risk *n* (%)	Unclear *n* (%)	High Risk *n* (%)
Sequence generation	7 (17.9%)	13 (33.3%)	19 (48.7%)
Allocation concealment	0 (0%)	13 (33.3%)	26 (66.7%)
Baseline comparability	32 (82.1%)	7 (17.9%)	0 (0%)
Random housing	0 (0%)	39 (100%)	0 (0%)
Blinding of investigators	0 (0%)	5 (12.8%)	34 (87.2%)
Blinding of outcome assessors	1 (2.6%)	38 (97.4%)	0 (0%)
Incomplete outcome data	34 (87.2%)	4 (10.3%)	1 (2.6%)

## Data Availability

No new data were created or analyzed in this study.

## Supplementary Materials

The following supporting information can be downloaded at: https://www.mdpi.com/article/10.3390/bioengineering13020169/s1, Supplementary Table S1. Extracted quantitative outcome data (means, SDs, group sizes, timepoints. Supplementary Table S2. Study-level SYRCLE risk-of-bias judgments (low/unclear/high) across all eight domains for each of the 39 included studies. Supplementary Table S3. Full extracted dataset of included studies: study identifiers, model parameters, intervention details, outcomes and numeric data (mean ± SD, *n*) with notes.
