# Supplementary material for: Scaffolds and Stem Cells Show Promise for TMJ Regeneration: A Systematic Review
Source: Bioengineering (Basel). 2026 Jan 29;13(2):169. doi: 10.3390/bioengineering13020169 (PMC12938102; doi:10.3390/bioengineering13020169)
Supplement: Supplementary file 1 [file bioengineering-13-00169-s001.zip › supplementary Table S2.pdf]

Supplementary Table S2. Study-level SYRCLE risk-of-bias judgments (low/unclear/high) across all eight domains for each of the 39 included studies.

SYRCLE Risk of Bias Assessment

Reference	Sequence generation	Allocation concealment	Baseline comparability	Random housing	Blinding of investigators	Blinding of outcome assessors	Incomplete outcome data	Selective reporting
22	High risk	High risk	Low risk	Unclear	High risk	Unclear	Low risk	Unclear
23	High risk	High risk	Low risk	Unclear	High risk	Unclear	Low risk	Unclear
24	High risk	High risk	Low risk	Unclear	High risk	Unclear	Low risk	Unclear
25	High risk	High risk	Low risk	Unclear	High risk	Unclear	Low risk	Unclear
26	High risk	High risk	Low risk	Unclear	High risk	Unclear	Low risk	Unclear
27	High risk	High risk	Low risk	Unclear	High risk	Unclear	Low risk	Unclear
28	High risk	High risk	Low risk	Unclear	High risk	Unclear	Low risk	Unclear
29	High risk	High risk	Low risk	Unclear	High risk	Unclear	Low risk	Unclear
30	High risk	High risk	Low risk	Unclear	High risk	Unclear	Low risk	Unclear
31	High risk	High risk	Low risk	Unclear	High risk	Unclear	Low risk	Unclear
32	High risk	High risk	Low risk	Unclear	High risk	Unclear	Low risk	Unclear
33	High risk	High risk	Low risk	Unclear	High risk	Unclear	Low risk	Unclear
34	High risk	High risk	Low risk	Unclear	High risk	Unclear	Low risk	Unclear

35	High risk	High risk	Low risk	Unclear	High risk	Unclear	Low risk	Unclear
36	High risk	High risk	Low risk	Unclear	High risk	Unclear	Low risk	Unclear
37	High risk	High risk	Low risk	Unclear	High risk	Unclear	Low risk	Unclear
38	High risk	High risk	Low risk	Unclear	High risk	Unclear	Low risk	Unclear
39	Unclear	Unclear	Unclear	Unclear	Unclear	Unclear	Unclear	Unclear
40	Low risk	Unclear	Low risk	Unclear	High risk	Unclear	Low risk	Unclear
41	Low risk	Unclear	Low risk	Unclear	High risk	Unclear	Low risk	Unclear
42	Unclear	High risk	Low risk	Unclear	High risk	Unclear	High risk/Unclear	Unclear
43	Unclear	Unclear	Unclear	Unclear	Unclear	Unclear	Unclear	Unclear
44	Unclear	High risk	Low risk	Unclear	High risk	Unclear	Low risk	Unclear
45	Unclear	High risk	Low risk	Unclear	High risk	Unclear	Low risk	Unclear
46	Unclear	Unclear	Unclear	Unclear	Unclear	Unclear	Unclear	Unclear
47	Low risk	Unclear	Low risk	Unclear	High risk	Unclear	Low risk	Unclear
48	High risk	High risk	Low risk	Unclear	High risk	Unclear	Low risk	Unclear
49	Unclear	High risk	Low risk	Unclear	High risk	Unclear	Low risk	Unclear
50	Unclear	Unclear	Unclear	Unclear	Unclear	Unclear	Unclear	Unclear
51	Low risk	Unclear	Low risk	Unclear	High risk	Unclear	Low risk	Unclear
52	Low risk	Unclear	Low risk	Unclear	High risk	Unclear	Low risk	Unclear
53	Low risk	Unclear	Low risk	Unclear	High risk	Unclear	Low risk	Unclear
54	Unclear	Unclear	Low risk	Unclear	High risk	Unclear	Low risk	Unclear
55	High risk	High risk	Unclear	Unclear	High risk	Unclear	Low risk	Unclear
56	Unclear	High risk	Unclear	Unclear	High risk	Unclear	Low risk	Unclear
57	Unclear	High risk	Low risk	Unclear	High risk	Unclear	Low risk	Unclear
58	Unclear	High risk	Unclear	Unclear	High risk	Unclear	Low risk	Unclear

59	Unclear	Unclear	Low risk	Unclear	High risk	Unclear	Low risk	Unclear
60	Low risk	Unclear	Low risk	Unclear	Unclear	Low/Unclear	Low risk	Unclear
